# Molecular Bases of Human Malformation Syndromes Involving the SHH Pathway: GLIA/R Balance and Cardinal Phenotypes

**DOI:** 10.3390/ijms222313060

**Published:** 2021-12-02

**Authors:** Yo Niida, Sumihito Togi, Hiroki Ura

**Affiliations:** 1 Center for Clinical Genomics, Kanazawa Medical University Hospital, Uchinada 9200293, Ishikawa, Japan; togi@kanazawa-med.ac.jp (S.T.); h-ura@kanazawa-med.ac.jp (H.U.); 2 Division of Genomic Medicine, Department of Advanced Medicine, Medical Research Institute, Kanazawa Medical University, Uchinada 9200293, Ishikawa, Japan

**Keywords:** SHH pathway, GLI family zinc finger protein, human malformation syndromes, polydactyly, primary cilium

## Abstract

Human hereditary malformation syndromes are caused by mutations in the genes of the signal transduction molecules involved in fetal development. Among them, the Sonic hedgehog (SHH) signaling pathway is the most important, and many syndromes result from its disruption. In this review, we summarize the molecular mechanisms and role in embryonic morphogenesis of the SHH pathway, then classify the phenotype of each malformation syndrome associated with mutations of major molecules in the pathway. The output of the SHH pathway is shown as GLI activity, which is generated by SHH in a concentration-dependent manner, i.e., the sum of activating form of GLI (GLIA) and repressive form of GLI (GLIR). Which gene is mutated and whether the mutation is loss-of-function or gain-of-function determine in which concentration range of SHH the imbalance occurs. In human malformation syndromes, too much or too little GLI activity produces symmetric phenotypes affecting brain size, craniofacial (midface) dysmorphism, and orientation of polydactyly with respect to the axis of the limb. The symptoms of each syndrome can be explained by the GLIA/R balance model.

## 1. Introduction

Hedgehog (HH) is a key morphogen involved in patterning during embryonic development; the HH pathway regulates pattern formation in various organs. HH is secreted from a specific group of cells and diffuses to the peripheral tissue, establishing a gradient that can induce or suppress the expression of developmental genes in a concentration-dependent manner. Consequently, HH concentration provides information on the positions of incipient body parts during development, assuring their correct orientation. HH was first identified by genetic screens in *Drosophila melanogaster* [1]. Shortly thereafter, three mammalian homologs—sonic hedgehog (SHH), Indian hedgehog (IHH) and desert hedgehog (DHH)—were discovered, and an evolutionarily conserved role for these molecules in developmental patterning established [2,3,4,5,6,7]. Of these, SHH is the most broadly expressed mammalian HH signaling molecule [8]. SHH is expressed in the zone of polarizing activity (ZPA) of the limb bud [3,7,9,10], and in those of the notochord and floor plate in the neural tube [11,12]. IHH regulates bone and cartilage development and is partly redundant with SHH [13], whereas DHH expression is largely restricted to gonads, including sertoli cells of testis and granulosa cells of ovaries [14,15,16]. Much research on the SHH pathway has been done, and due to the general cross-species conservation of function, studies using animal models were instrumental in understanding its basic functions. To date, the roles of many genes belonging to the SHH pathway have been elucidated. However, the phenotypes of hereditary malformation syndromes in humans do not always recapitulate those of genetically modified animal models due to species-specific characteristics of the HH pathway. It is therefore necessary to characterize and classify the human phenotypes caused by mutations SHH pathway genes, to better understand the functions of these genes in human development. Human traits displaying Mendelian inheritance are summarized in the Online Mendelian Inheritance in Man database (OMIM, https://omim.org/; accessed on 8 November 2021), and terms related to morphological abnormalities are defined by the element of morphology [17,18,19,20,21,22,23,24]. Individual human morphological abnormalities can be scientifically classified by the molecular mechanism in which they have their origin. In the case of syndromes associated with the SHH pathway, there is a correlation between morphological abnormalities and the net effect of the output level of the SHH pathway: excess and deficiency of output each result in symmetrical morphological abnormalities. In this review, we first expound the basic mechanism of the SHH pathway, which is supported by a cellular structure known as the primary cilium. Next, we classify the phenotypes of human malformation syndromes due to mutations in the major constituent genes of the SHH pathway.

## 2. The SHH Pathway

### 2.1. SHH Acts by Establishing a Morphogen Gradient

**Figure 1:**
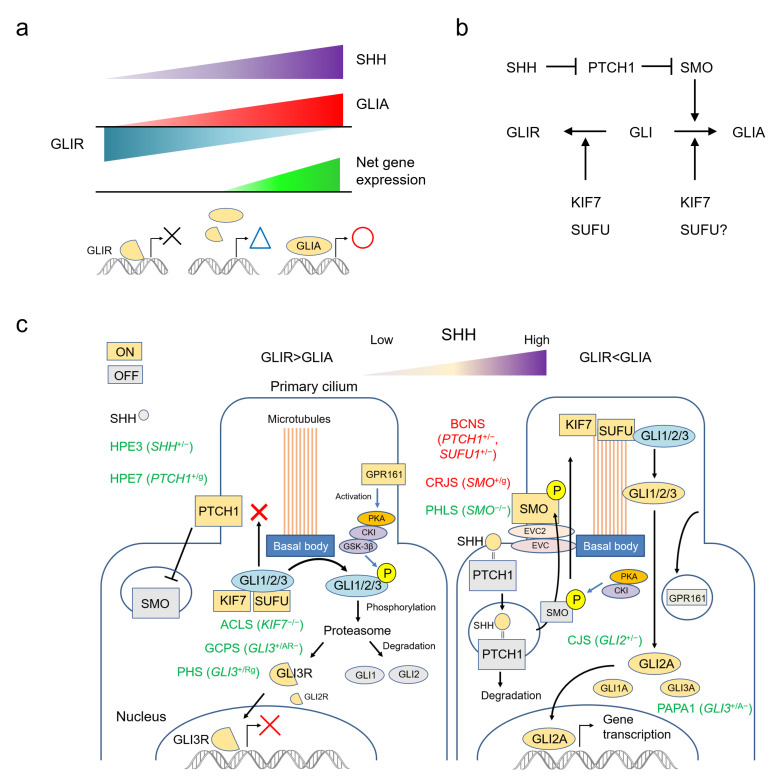
The SHH pathway and human malformation syndromes. (**a**) The output of the SHH pathway is expressed as a balance between GLIA and GLIR, fluctuating continuously rather than according to an all-or-nothing rule. (**b**) Simplified molecular circuit of the pathway. (**c**) Schematic representation of the relationship between the function of each molecule and the primary cilium, and associated syndromes. Each molecule changes its function by migrating to a specific site of the primary cilium. ACLS: acrocallosal syndrome; BCNS: basal cell nevus syndrome; CJS: Culler-Jones syndrome; CRJS: Curry-Jones syndrome; GCPS: Greig cephalopolysyndactyly syndrome; HPE: holoprosencephaly; PAPA1: polydactyly, postaxial, types A1 and B; PHLS: Pallister-Hall-like syndrome; PHS: Pallister-Hall syndrome.

SHH is one of the most important morphogens in animals, and is involved in the pattern formation of many organs, including limbs and the ventral midline structure of the central nervous system [25]. GLI family zinc finger proteins are the only known transcriptional effectors of the sonic hedgehog (SHH) signalling pathway; they control human embryonic development by regulating transcription of a group of target genes [25]. The vertebrate *Gli* gene family is thought to be derived from duplications of a single ancestral chordate gene [26], and four *GLI* genes—*GLI1* (12q13.3), *GLI2* (2q14.2), *GLI3* (7p14.1) and *GLI4* (8q24.3)—have been identified in humans [27,28]. GLI acts as a transcriptional enhancer (GLIA) in the presence of SHH, but suppresses the same target genes in the absence of SHH (GLIR). The output of the SHH pathway is expressed as a balance between GLIA and GLIR. The higher the SHH concentration, the higher the GLIA production; the lower the SHH concentration, the higher the GLIR production. The resulting net expression of target genes fluctuates continuously, and does not obey an all-or-nothing rule (Figure 1a).

### 2.2. The SHH Pathway and The Primary Cilium

Recently, the functions and mechanisms of the SHH pathway have been well-summarized in several reviews [13,25,29,30,31]. To put it very simply based on these references, SMO is the key molecule that activates GLI into GLIA. PTCH1 constitutively suppresses the action of SMO, but this suppression is removed by SHH. As a result, SMO is activated in the presence of SHH and GLIA production is increased. Conversely, SMO remains suppressed in the absence of SHH, and GLI is converted to GLIR. KIF7 is involved in both activation and inhibition of GLI. SUFU mainly works to suppress GLI, but in some cases it may be involved in GLI activation (Figure 1b).

The functions of molecules in the SHH pathway depend on the structure of the primary cilium (Figure 1c). This solitary organelle emanates from the cell surface of most mammalian cell types during growth arrest. Increasing evidence suggests that primary cilia are key coordinators of signaling pathways—including PDGF, Hedgehog, Wnt and mechano-signaling—during development and in tissue homeostasis [32].

The SHH signaling pathway can be summarized as follows: in the absence of SHH, the PTCH1 constitutively present in the primary cilium suppresses SMO. KIF7 and SUFU bind to the GLI protein, keeping it at the basal body and prohibiting entry of GLI into the cilium. Here, GLI is phosphorylated by the PKA, CKI, GSK-3β complex activated by GPR161, and degraded by the proteasome. Under these conditions, GLI1 and GLI2 are almost completely degraded, but GLI3 is converted to GLI3R and functions as an inhibitory transcription factor. When SHH is present, it binds to PTCH1 and promotes its degradation. Freed of PTCH1-mediated suppression, SMO is phosphorylated and translocated to the primary cilium. SUFU-bound GLI protein aggregates on the cilium tip, where GLI is released by the action of KIF7. Free GLI is then activated intracellularly. GLI2A is thought to be the most important activator, with the role of GLI3A small and that of GLI1A even smaller (Figure 1c) [13,25,29,30,31].

## 3. Human Development and Malformations Related to the SHH Pathway

Hedgehog is not the only morphogen involved in fetal development. During embryogenesis, a number of key signaling pathways—Fgf, Hedgehog, Wnt, TGFß, and Notch being among the most important—work together in harmony, appearing repeatedly at different times and in different regions in the embryo and eliciting diverse cellular responses [33]. SHH plays a central role in the formation of midline structures [34], the determination of the number of neurons in the cerebral cortex [35], the migration of cranial neural crest cells (CNCCs) [36], and the determination of the anterior-posterior axis of the digits [37]. Consequently, the following anomalies are particularly important when considering the symptoms of human malformation syndromes caused by genetic defects in the SHH pathway.

### 3.1. Brain Morphology and Size (Holoprosencephaly and Micro/Macro-Cephaly)

Holoprosencephaly (HPE) is a brain malformation in which the prosencephalon or embryonic forebrain fails to divide into two separate lobes between the third and fourth weeks of gestation. This process results in varying degrees of lack of separation of the cerebral hemispheres, classically classified alobar, semilobar and lobar in decreasing order of severity [38]. Mutations in SHH were the first genetic causes of human holoprosencephaly to be identified [39]. SHH is present throughout the axial mesendoderm of the early embryo, including in the prechordal plate, and plays an essential role in establishing subdivision of the eyefield and bilateral patterning of the ventral forebrain [12,40]. Other genes associated with or independent of the SHH pathway, and chromosomal abnormalities (most frequently trisomy 13), have more recently been described as causing holoprosencephaly [41,42].

SHH signaling also defines brain size. SHH signaling endogenously regulates the number of embryonic and postnatal mouse neocortical cells with stem cell properties, and controls precursor proliferation in a concentration-dependent manner [43]. Central nervous system–specific deletion of the gene encoding the essential adherens junction protein aE-catenin causes abnormal activation of the hedgehog pathway, resulting in shortening of the cell cycle, decreased apoptosis, and cortical hyperplasia in mice [44]. During the development of the neocortex, SHH is expressed at low levels in the neural stem/progenitor cells as well as in mature neurons in the dorsal telencephalon. It seems that high-level SHH signaling in the ventral telencephalon is essential for ventral specification, while low-level SHH signaling in the dorsal telencephalon plays an important role in the fine-tuning of cell cycle kinetics. SHH signaling regulates cell cycle kinetics of radial glial cells and intermediate progenitor cells, thereby maintaining the proliferation, survival and differentiation of neurons in the neocortex [45]. The expansion of outer radial glia (oRGs, also called basal RGs) and intermediate progenitor cells (IPCs) in the cortical subventricular zone has played a key role in the evolutionary expansion and folding of the neocortex. SHH signaling activity in fetal neocortex is stronger in humans than in mice, and blocking SHH signaling decreases oRGs in human cerebral organoids [46]. These data strongly suggest that the activity of the SHH pathway determines the size of brain. Even in human malformation syndromes, increased activity of the SHH pathway is thought to be associated with macrocephaly (large brain), and decreased activity is associated with microcephaly (small brain).

### 3.2. Craniofacial (Midface Anormalies)

SHH is a key signaling factor in the regulation of craniofacial skeleton development in vertebrates, operating within numerous tissues in the craniofacial primordia to spatiotemporally regulate the formation of the face and jaws [36]. In order to form the facial bones, CNCCs migrate ventrally into the frontonasal prominence (FNP), as well as the first, second, third and fourth pharyngeal arches (PA1-4) [47]. Craniofacial defects caused by SHH deficiency occur even with correct ventralization and development of the floor plate of the neural tube, indicating a specific regulatory role for SHH within the pharyngeal arches themselves, rather than these defects being a secondary consequence of neural tube defects [48]. SHH is a critical factor for development and survival of CNCCs within pharyngeal arches, which eventually colonize the facial prominences [49,50]. As a result of CNCC death, mice lacking *Shh* exhibit failure of anterior facial structure formation [40]. Specifically, *Shh*^−/−^ mice demonstrate normal early patterning of PA1 until embryonic day 9.5 (E9.5); however, within 24 h, PA1 is greatly reduced in size, indicative of global first arch atrophy [51].

**Figure 2:**
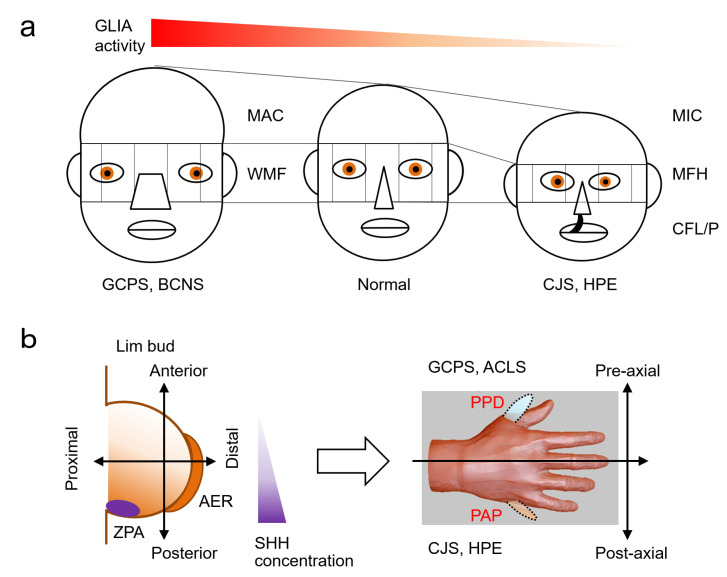
Human malformation syndromes and the SHH pathway. (**a**) Excessive SHH activity is associated with macrocephaly and midface hyperplasia, while underactivity is associated with microcephaly and midface hypoplasia. (**b**) The location of polydactyly depends on the anterior-posterior axis of the embryonic limb bud formed by the SHH concentration gradient. Abnormalities in low SHH concentration areas lead to preaxial polydactyly, and conversely, problems in high SHH concentration areas lead to postaxial polydactyly. ACLS: Acrocallosal syndrome; AER: apical ectodermal ridge; BCNS: Basal cell nevus syndrome; CFL/P: cleft lip and/or palate; GCPS: Greig cephalopolysyndactyly syndrome; CJS: Culler-Jones syndrome; HPE: Holoprosencephaly; MAC: macrocephaly; MFH: midface hypoplasia; MIC: microcephaly; PPD: preaxial polydactyly; PAP: postaxial polydactyly; SHH: sonic hedgehog; WMF: wide mid face; ZPA: zone of polarizing activity.

In human craniofacial malformations, insufficient SHH signaling activity presents with “midface hypoplasia”, including cleft lip/palate, flat nose with nasal septum hypo- or agenesis, and orbital deformation with facial asymmetry. These features resemble to facial appearance of holoprosencephaly (HPE), but can occur independently rather than as part of a syndrome [52]. This HPE-like facial dysmorphia without obvious brain anomalies is called microform HPE or HPE-like syndrome [53,54]. Usually, it is associated with microcephaly and hypotelorism (the distance between the eyes is decreased). Interestingly, these craniofacial features are reversed in malformation syndromes associated with excess SHH signaling activity. Namely, these include macrocephaly, frontal bossing, and hypertelorism (increased distance between the eyes), all of which are accompanied by a general broadening of midline facial structures [55] (Figure 2a).

### 3.3. Limbs and Digits (Polydactyly and Syndactyly)

Sonic hedgehog (*Shh*) is expressed in the zone of polarizing activity (ZPA), a small group of mesenchymal cells at the posterior margin of the vertebrate limb bud. It has been shown that SHH signaling can specify the antero-posterior axis (e.g., thumb to little finger) of the limb in both a concentration- (paracrine) and time-dependent (autocrine) fashion [37]. Unsurprisingly, mutations in genes in the SHH pathway cause malformation of digits, including polydactyly (extra fingers or toes) and syndactyly (some or all of the fingers or toes wholly or partly fused). Both poly- and syndactyly may be present at the same time, or may occur independently. A plethora of polydactyly classification methods has been reported in the medical literature, which deal with the heterogeneity of polydactyly in various ways. However, polydactyly can be broadly classified into two major types: pre-axial polydactyly (extra digits on the thumb or hallux side) and post-axial polydactyly (extra digits on the little finger or small toe side) [56]. Because the concentration of SHH determines the antero-posterior axis, when the SHH pathway abnormality occurs in the region where the SHH concentration is low (anterior area), it tends to be pre-axial polydactyly, and when it occurs in the region where the Shh concentration is high (posterior area), it tends to be post-axial polydactyly (Figure 2b).

### 3.4. Neoplasia (Basal Cell Carcinoma, Medulloblastoma)

In addition to embryonic morphogenesis, the SHH pathway is required for somatic stem cell maintenance. The HH pathway is mostly inactive or poorly active in the adult organism. If necessary, it can be activated—in wound healing, for example [57,58]. Overactivity of the SHH pathway is involved in the development of cancer. Basal cell nevus syndrome is an example: a typical hereditary cancer syndrome where basal cell carcinomas and medulloblastomas develop [59]. On the other hand, abnormal activation of the SHH pathway has also been found in sporadic cancers. In addition to basal cell carcinoma and medulloblastoma, it is known to be associated with pancreatic, breast, colon, ovarian, and small-cell lung carcinomas [58]. Several HH signaling pathway inhibitors, such as vismodegib and sonidegib, have been developed for cancer treatment [60,61,62].

## 4. Human Malformation Syndromes Caused by Major Genes in the SHH Pathway

**Figure 3:**
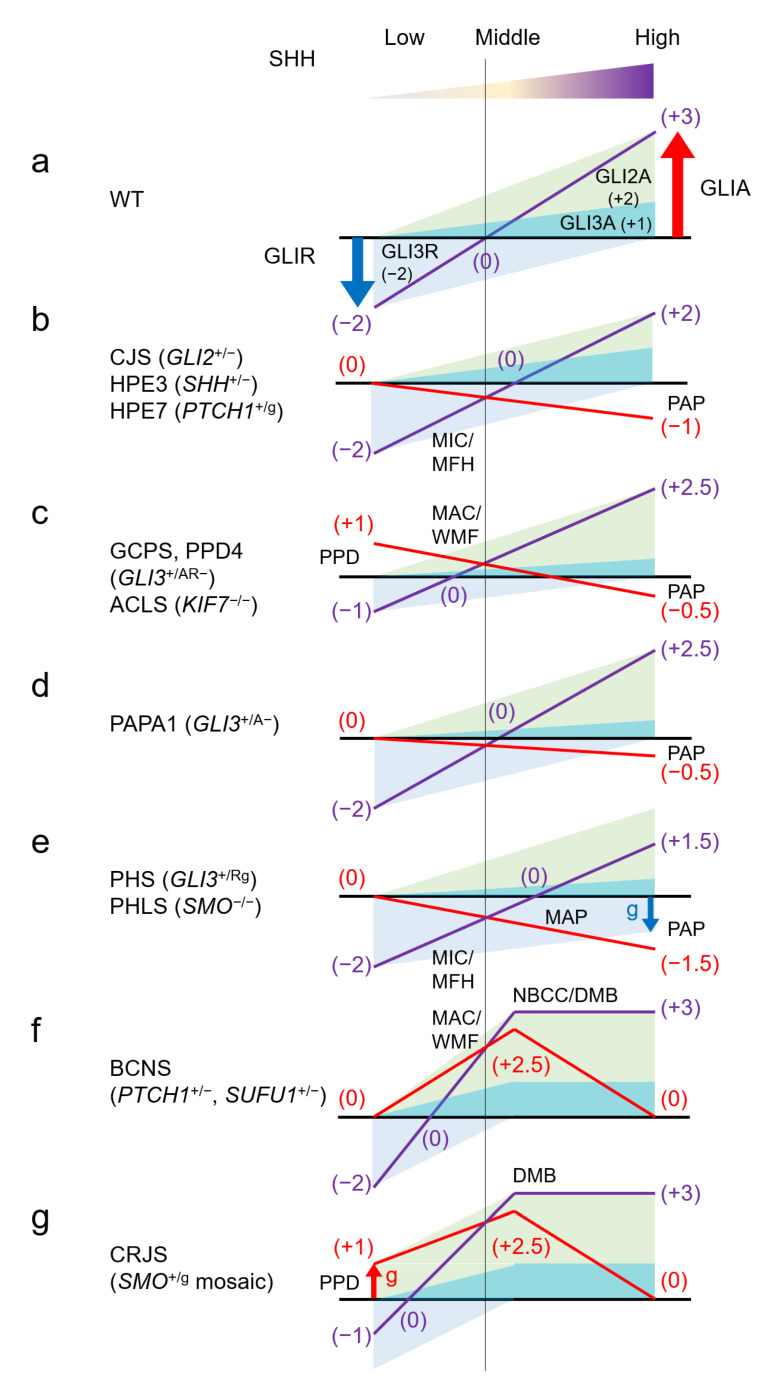
Human malformation syndromes and the GLIA/R balance model. Assume that suppression of GLI3R is −2 and activity of GLI2A and GLIA3 is 2 and 1, at maximum and minimum SHH concentrations, respectively. The output balance of GLIA/R is assumed to fluctuate linearly from zero to saturation of SHH concentration. The effect of the sum of GLIA and GLIR in each syndrome is shown by the purple line, and the deviation from the wild type (**a**) is shown by the red line. The actual amount ratio of GLIA and GLIR is unknown and may differ depending on the tissue, but it should be noted that the relationship between the direction of deviation of GLIA/R balance from the wild type and correlation to SHH concentration in each syndrome is unchanged. Preaxial polydactyly correlates with an overbalance in the low SHH region (thumb side) (**c**,**g**), and postaxial polydactyly correlates with an underbalance in the high SHH region (little finger side) (**b**–**e**). Microcephaly/midface hypoplasia correlates with an underbalance in the lower-intermediate SHH region (**b**,**e**), and macrocephaly/wide midface correlates with an overbalance (**c**,**f**). Also, neoplasia correlates with an overbalance in the intermediate SHH region (**f**,**g**). ^A^: active form of GLI; ^AR^: both active and repression form of GLI; ACLS: acrocallosal syndrome; BCNS: basal cell nevus syndrome; CJS: Culler-Jones syndrome; CRJS: Curry-Jones syndrome; DMB: desmoplastic medulloblastoma; g: gain of function mutation; GCPS: Greig cephalopolysyndactyly syndrome; HPE: holoprosencephaly; MAC: macrocephaly; MAP: mesoaxial polydactyly; MFH: midface hypoplasia; MIC: microcephaly; NBCC: nevoid basal cell carcinoma; PAP: postaxial polydactyly; PPD: preaxial polydactyly; PAPA1: polydactyly, postaxial, types A1 and B; PHLS: Pallister-Hall-like syndrome; PHS: Pallister-Hall syndrome; ^R^: repression form of GLI;WMF: wide midface; WT: wild type.

This section summarizes the major human malformation syndromes involving the SHH pathway, by gene and mutation type. “Loss of function” (LOF) refers to a mutation in which a gene product (protein) is not produced or does not function if it is produced. On the other hand, “gain of function” (GOF) refers to a mutation in which a gene product is produced that functions abnormally. It does not matter whether its function is the same as or vice versa of the function that a normal gene product gives to the SHH pathway. The OMIM registration number of each gene and disease are listed. Refer the OMIM FAQs (https://omim.org/help/faq#1_3; accessed on 8 November 2021) for the meaning of the symbols before the numbers. Core clinical features of each syndrome are summarized in Table 1, and the effects of each gene mutation on GLIA/R balance are shown in Figure 3. Many of these diseases are autosomal dominant, and fetal lethality is possible in those for which there are no reports of human genetic disorders due to homozygous LOF or GOF mutations. Similarly, some of these GOF mutations are only known to occur as somatic mosaics; in these cases, germline heterozygous mutations are also likely to cause embryonic lethality.

### 4.1. GLI2 (GLI Family Zinc Finger 2) *165230

#### 4.1.1. Culler-Jones Syndrome; CJS #615849

CJS is caused by heterozygous LOF mutations in *GLI2*. This autosomal dominant disorder is characterized by hypoplasia of the midface and the pituitary gland—which is in turn associated with anterior pituitary dysfunction—and/or postaxial polydactyly (PAP). The disorder shows incomplete penetrance and variable expressivity: some patients have midline facial defects and developmental delays [63,64,65]. The patient manifests short stature due to growth hormone deficiency, and occasionally, a delay of puberty. Some patients manifest polydactyly, mostly postaxial without syndactyly, and variable degrees of intellectual and socio-behavioural disabilities. Genotype-phenotype correlations do not appear to fit the typically expected pattern: missense mutations are not always milder than truncating mutations, and point mutations are not always milder than chromosomal microdeletions [66]. Although patients with large chromosomal deletions tend to have complicating visceral malformations, such as congenital heart defects, heterotaxy, and urogenital defects, the phenotypic spectra of point mutations and chromosomal deletions generally overlap and can be difficult to distinguish. Intellectual disability in particular is highly unpredictable. Notably, a patient with a 20 Mb deletion had normal intelligence [67], and occasionally, several microdeletions are inherited from parents with normal or minimal phenotypes [68].

According to the GLIA/R output balance regulation mechanism of the SHH pathway shown in Figure 1c, GLIR is generated in an environment with a low SHH concentration, and GLIA is produced in an environment with a high SHH concentration (Figure 3a). Since the main component of GLIR is GLI3, *GLI2* haploinsufficiency does not affect the regions where the SHH concentration is low. On the other hand, since the main component of GLIA is GLI2, haploinsufficiency of *GLI2* significantly decreases GLIA in the regions where SHH concentration is high. Additionally, even in the regions where the SHH concentration is intermediate, GLIA activity is lower than normal (Figure 3b). This abnormal GLIA/R balance is corelated with the development of the above-mentioned malformations that occur in CJS.

#### 4.1.2. Holoprosencephaly 9; HPE9 #610829

HPE9 and CJS belong to a continuous phenotypic spectrum, with the relatively mild form referred as CJS and the severe form known as HPE9. HPE9 is characterized by a wide phenotypic spectrum of brain developmental defects, with or without overt forebrain cleavage abnormalities (holoprosencephaly). To date, many case reports and reviews of human *GLI2* defects associated with HPE9 have been made [69,70,71], and the actual phenotypic spectra of these syndromes have been clarified [66].

### 4.2. GLI3 (GLI Family Zinc Finger 3) *165240

#### 4.2.1. Greig Cephalopolysyndactyly Syndrome; GCPS #175700

GCPS is caused by heterozygous LOF mutations in *GLI3*. This autosomal dominant disease is characterised by polysyndactyly, macrocephaly, and correlated facial dysmorphisms (frontal bossing, ocular hypertelorism, and down-slanted palpebral fissures). Typical polysyndactyly features are bilateral preaxial polysyndactyly (PPD) of the feet, broad thumb, or PPD with simple syndactyly of other digits of the hands. In addition, PAP in the hands may present [72]. GCPS can result from point mutations in *GLI3* or by contiguous gene deletion syndrome of 7p13 (GCPS-CGS). Most point mutations result in intellectually normal patients and manifest with variable grades of polysyndactyly [73]. In contrast, GCPS-CGS is always associated with moderate to severe mental retardation and a consistent PPD of the big toe [74].

The genotype–phenotype correlations of *GLI3* point mutations are well-established [72,75]. The N-terminal part of the GLI3 protein contains the zinc finger domain (ZFD; amino acid residues, AA 462–645). Truncating mutations upstream or within the ZFD abolish DNA-binding activity and result in a total loss of protein function if translated. Typically, these mutations result in GCPS. Considering the GLIA/R balance, functional loss of one of the *GLI3* alleles halves the amount of GLIR produced in the regions where the SHH concentration is low. Consequently, GLI activity is higher than normal in these regions. GLI3A also decreases in the regions where SHH concentration is high, but the effect is minimal because the main component of GLIA is GLI2. In the regions with intermediate SHH concentration, GLI activity is also higher than normal (Figure 3c). These perturbations in GLIA/R balance can explain why GCPS presents predominantly with preaxial polydactyly (though occasionally with postaxial) and macrocephaly.

#### 4.2.2. Polydactyly, Preaxial, Type IV; PPD4 #174700

PPD4 and GCPS belong to a continuous phenotypic spectrum. The cranio-facial features of GCPS may be minimal or unclear in some patients, who can then be diagnosed with PPD4 if their syndrome is limited to preaxial polydactyly [76,77].

#### 4.2.3. Polydactyly, Postaxial, Types A1 and B; PAPA1 #174200

Heterozygous LOF mutations in *GLI3* can cause isolated polydactyly. Mutation of the C-terminal part of the GLI3 protein, which contains the transactivating domains that mediate the activator function of the protein, result in a phenotype that varies from typical GCPS to PPD4, or PAPA1 [isolated PAP with well-formed (type A1) or rudimentary (type B) digit] [75,78]. Precise genotype-phenotype correlations in these cases have not been established. If the mutated protein is expressed, it can convert to GLI3R normally with low SHH levels, but GLI3A activity is decreased under high SHH concentrations. This type of mutation may manifest as PAPA1 (Figure 3d).

#### 4.2.4. Pallister-Hall Syndrome; PHS #146510

PHS is caused by heterozygous GOF mutations in *GLI3*. Truncating mutations in the middle part of the protein generate a ZFD-only version of GLI3 that does not include the transactivating domains 1 and 2 (TA2, AA 1044–1322; and TA1, AA 1376–1580) located on the C-terminal end of the protein. This mutant protein acts similarly to GLI3R, and it cannot be converted to GLI3A, leading to an extreme abundance of GLI3R activity and decoupling it from SHH signaling.

PHS is characterised by midface hypoplasia similar to or more severe than CJS, postaxial or mesoaxial (near the center of the axis) polydactyly, and other central structure abnormalities, including hypothalamic hamartoma, bifid epiglottis or laryngeal cleft, and pulmonary segmentation anomalies.

In PHS, one allele of *GLI3* produces constantly GLI3R by mutation, so the GLIA/R balance is generally inclined toward GLIR. However, in regions where the SHH concentration is very low, there is no excess or deficiency compared to normal. Therefore, in PHS, symptoms related to insufficient GLI activity appear strongly, but symptoms due to excess GLI activity such as GCPS do not appear. For polydactyly, GLIA deficiency affects the more central region of the anterior-posterior axis, resulting in mesoaxial polydactyly, but preaxial polydactyly does not occur (Figure 3e).

### 4.3. SHH (Sonic Hedgehog Signalling Molecule) *600725

Mutations in the *SHH* gene itself also contribute to human malformation syndromes, as a series of related symptoms. All of these syndromes are caused by LOF mutations in one allele of *SHH*, and are classified as autosomal dominant due to haploinsufficiency.

#### 4.3.1. Holoprosencephaly 3; HPE3 #142945

HPE3 presents with varying degrees of holoprosencephaly-, microcephaly- and midface hypoplasia-related symptoms. Nanni et al. presented a panel of 12 photographs illustrating the range of severity in holoprosencephaly resulting from mutations in the *SHH* gene [79].

#### 4.3.2. Microphthalmia, Isolated, with Coloboma 5; MCOPCB5 #611638

Schimmenti et al. reported a family with a heterozygous 24 bp deletion in *SHH*. Proband is an 8-month-old boy with bilateral microphthalmia with bilateral iris, and right chorioretinal and left uveoretinal coloboma (missing of any of eye structure). The boy had no stigmata of holoprosencephaly or other malformations. Incomplete expression of the *SHH* mutation was observed, as his mother, who had unilateral iris and uveoretinal coloboma, and 3 unaffected family members, all carried the same deletion [80].

#### 4.3.3. Single Median Maxillary Central Incisor; SMMCI #147250 (SMMCI Syndrome Included)

Single median maxillary central incisor (SMMCI) is one of the symptoms associated with dysplasia of the median facial structure. It is often associated with the holoprosencephaly spectrum, but can also occur as an isolated malformation [81,82].

### 4.4. PTCH1 (Patched 1) *601309

#### 4.4.1. Basal Cell Nevus Syndrome; BCNS #109400

Heterozygous LOF mutations in *PTCH1* cause basal cell nevus syndrome (BCNS), also known as Gorlin syndrome. BCNS is characterized by numerous basal cell cancers of the skin, keratocysts of the jaws, palmar and plantar pits, ovarian fibromas, medulloblastomas, and various malformations. Craniofacial manifestations include macrocephaly, broad facies, frontal bossing, hypertelorism and broad nasal root (Figure 2a). It is also associated with calcification of the falx cerebri, rib and vertebral abnormalities, cleft lip or cleft palate, and cortical defects of bones, but not polydactyly or syndactyly [59,83]. Kimonis et al. tabulated major and minor BCNS criteria, and defined diagnosis of BCNS in terms of the presence of two major or one major and two minor criteria [59]. Biallelic disruption of *PTCH1* (second hit mutation) is reported in basal cell carcinomas [84] and ovarian leiomyomas [85] in patients with BCNS. BCNS is also caused by heterozygous LOF mutations in *PTCH2* [86] and *SUFU* (see Section 4.6.1).

PTCH1 is a receptor of SHH; the binding of PTCH1 to SHH de-represses SMO, leading to downstream activation of GLI. When the amount of protein is halved by loss of one *PTCH1* allele, the amount of SHH required for maximal GLI activation is also halved compared to the wild type. Therefore, there is no difference from wild type in regions where SHH concentration is at minimum or maximum, but activation of GLI is strongly enhanced in regions where the amount of SHH is intermediate or relatively low, compared to wild type (Figure 3f). This explains the presentation of macrocephaly and wide midface and the absence of polydactyly, and suggests an association with the development of cancer.

Cleft lip/palate is also observed in some patients with BCNS. In mice, loss of *Ptch1* function in cranial neural crest cells (which relieves SMO inhibition and leads to constitutive activation of the HH pathway) has been shown to cause mid-facial expansion, which culminates in cleft lip as well [87]. It is thought that the same mechanism applies in humans [55]. Therefore, cleft lip/palate can be caused by both less or excess of SHH pathway activity.

#### 4.4.2. Holoprosencephaly 7; HPE7 #610828

Heterozygous GOF mutations in PTCH1 cause HPE with varying degrees of symptoms (HPE7) [88,89]. Missense mutations that produce a PTCH1 protein with reduced SHH binding capacity or reduced capacity for signal transduction attenuate SHH pathway activation. Such mutations in one of the *PTCH1* alleles have the same effect as LOF mutations that halve functional SHH protein (HPE3) (Figure 3b).

Also, mothers and children with microcephaly and developmental delay due to a ~360 Kb duplication on chromosome 9q22.32, which includes *PTCH1*, have been reported [90]. In this case, *PTCH1* becomes 3 copies and the SHH pathway is strongly suppressed.

### 4.5. SMO (Smoothened, Frizzled Class Receptor) *601500

#### 4.5.1. Pallister-Hall-Like Syndrome; PHLS #241800

PHLS caused by homozygous LOF mutations of *SMO*. This autosomal recessive disease is characterized by microcephaly, facial dysmorphism associated with midface hypoplasia, and postaxial polydactyly with variable expressivity. Patients also exhibit hypothalamic hamartoma, cardiac and skeletal anomalies, and Hirschsprung disease [91,92]. Because SMO is the main transducer of positive SHH signalling, loss of SMO results in a broad reduction in GLI activity in regions with intermediate to high concentration of SHH—much more severe than in *GLI2* or *SHH* haploinsufficiency. The profile of GLIA/R balance is expected to be similar to that of PHS (Figure 3e).

#### 4.5.2. Curry-Jones Syndrome; CRJS #601707

CRJS caused by GOF mutations in *SMO* with somatic mosaicism. Twigg et al. studied tissue samples from 10 unrelated patients with Curry-Jones syndrome, and identified somatic mosaicism for an identical missense mutation in *SMO* in all cases: NM_005631.4:c.1234C>T p.(Leu412Phe) [93]. Curry-Jones syndrome is a multisystem disorder characterized by patchy skin lesions (hypopigmented streaky lesions), polysyndactyly, diverse cerebral malformations, unicoronal craniosynostosis, iris colobomas, microphthalmia, and intestinal malrotation with myofibromas or hamartomas.

The p.Leu412Phe mutation is thought to constitutively activate SMO and explains the development of preaxial polydactyly and medulloblastoma in the GLIA/R balance model (Figure 3g). In addition, CRJS is accompanied by asymmetry of the skull and orbit due to mosaic mutation. Skin symptoms and colorectal symptoms not found in other SHH pathway syndromes are observed, which are also considered to be the result of GOF mosaic mutations in *SMO*.

### 4.6. SUFU (SUFU Negative Regulator of Hedgehog Signaling) *607035

#### 4.6.1. Basal Cell Nevus Syndrome; BCNS #109400

As with *PTCH1*, heterozygous LOF mutations in *SUFU* also cause BCNS [94,95,96]. As the name implies, SUFU is a suppressor of the SHH pathway. Halving the amount of functional SUFU protein results in GLI activation occurring at lower concentrations of SHH and is expected to exhibit a profile similar to *PTCH1* haploinsufficiency (Figure 3f).

#### 4.6.2. Medulloblastoma, Desmoplastic; MDB #155255

*SUFU* haploinsufficiency also known to cause familial or sporadic desmoplastic medulloblastoma [97,98,99,100]. Familial cases showed incomplete penetrance, and according to Brugières et al. [101], 7 of 25 mutation carriers in 2 families developed medulloblastoma.

#### 4.6.3. Meningioma, Familial, Susceptibility to #607174

Aavikko et al. reported a family of five meningioma-affected siblings, four of whom had multiple tumors. A heterozygous *SUFU* missense mutation, NM_016169.4:c.367C>T p.(Arg123Cys), segregated with the meningiomas in the family, and functional analyses showed that the activity of the altered SUFU was significantly reduced. Also, all seven meningiomas analyzed displayed loss of the wild type allele according to the classic two-hit model for tumor-suppressor genes [102].

#### 4.6.4. Joubert Syndrome 32; JBTS32 #617757 and Corelated Conditions

In mice, homozygous targeted disruption of *Sufu* led to embryonic lethality at mid gestation (~E9.5) with cephalic and neural tube defects [103,104]. Similarly, there have been no reports of complete functional loss of both alleles of *SUFU* in humans, and this genotype is expected to be embryonic lethal [105,106]. However, Mori et al. reported that hypomorphic (partial loss of gene function) recessive variants in *SUFU* cause Joubert syndrome with cranio-facial and skeletal defects (JBTS32) in two families [105]. These patients have homozygous *SUFU* missense variants: either NM_016169.4:c.1217T>C p.(Ile406Thr) or c.527A>G p.(His176Arg). Functional studies on fibroblasts and cell lines showed that the mutant proteins were less stable and more rapidly degraded than wild type SUFU, and their ability to bind GLI3 and promote GLI3R was impaired, while their ability to bind GLI1 was unaltered. The affected children presented congenital ataxia and cerebellar vermis hypoplasia with elongated superior cerebellar peduncles (mild “molar tooth sign”), typical cranio-facial dysmorphisms (macrocephaly, hypertelorism, depressed nasal bridge, frontal bossing), and postaxial polydactyly.

These craniofacial features are similar to BCNS and can be explained as a result of increased GLI activity due to *SUFU* mutations. However, postaxial polydactyly indicates a decrease in GLI activity, and molar tooth sign indicates atrophy of the brain stem and cerebellum, which also suggests a decrease in GLI activity.

Mice with Cre recombinase (Cre)-mediated *SuFu* inactivation targeted to the cerebellum display abnormal mid-hindbrain morphology with cerebellar atrophy, via delayed differentiation and abnormal migration of major cerebellar cell types. Expression of a *Gli3* allele encoding constitutive GLI3R in *SuFu*-deficient mice largely rescues abnormal cerebellar patterning. Accordingly, *SuFu* controls cerebellar patterning and cell differentiation in a GLI3R-dependent manner [107]. SUFU also plays a pivotal role in controlling GLI protein levels, as it protects full length GLI2 and GLI3 proteins from SPOP (speckle-type POZ protein) mediated ubiquitination and complete degradation by the proteasome [108]. These data indicate that a significant decrease in SUFU function causes a decrease GLI protein due to accelerated degradation, which may lead to a decrease in production of both GLIR and GLIA. This may explain postaxial polydactyly and cerebellar atrophy in JBTS32.

It was recently found that haploinsufficiency of *SUFU* (heterozygous LOF mutation) also presents with congenital ocular motor apraxia [109] and neurodevelopmental delay with the mild Joubert syndrome phenotype [106]. These phenotypes are a continuous spectrum and show incomplete penetrance in familial cases. In many patients, macrocephaly is also present.

As mentioned above, *SUFU* haploinsufficiency presents with two distinct disease spectra. One is hereditary cancer syndromes and the other is Joubert syndrome-related neurodevelopmental disease. Although craniofacial abnormalities are common to both, there are no patients with comorbid malignant tumors and cerebellar symptoms. It is not clear what causes this discrepancy. Both syndromes are caused by LOF *SUFU* mutations, and some of these mutations occur both in patients with neurodevelopmental phenotypes and in patients with cancer [106], but these phenotypes do not overlap.

### 4.7. KIF7 (Kinesin Family Member 7) *611254

#### 4.7.1. Acrocallosal Syndrome; ACLS #200990

Acrocasllosal syndrome (ACLS) is an autosomal recessive condition caused by biallelic *KIF7* LOF mutations. Unlike in GCPS caused by *GLI3* haploinsufficiency, heterozygous *KIF7* LOF mutations do not affect phenotype. That is, in humans, *KIF7* is haplosufficient. Patients with ACLS manifest macrocephaly, preaxial or postaxial polydactyly, and mental retardation; ACLS patients are virtually indistinguishable from GCPS-CGS patients by their phenotype alone [110].

As shown in Figure 1, KIF7 localizes GLI to the base of the primary cilium to produce GLIR. The loss of KIF7 is thought to destabilize this localization and partially reduce GLIR production. As a result, GLI activity is higher than normal in regions where SHH concentration is low. On the other hand, KIF7 is also thought to assist in releasing GLI from SUFU at the top of primary cilium, and in this context promotes GLIA production. Therefore, loss of KIF7 partially suppresses GLIA production in regions with high SHH concentration. The effect of KIF7 loss on overall GLIA/R balance is similar to that of *GLI3* haploinsufficiency, explaining why the clinical manifestations of ACLS and GCPS are similar (Figure 3c).

#### 4.7.2. Al-Gazali-Bakalinova Syndrome; AGBK #607131

Al-Gazali-Bakalinova syndrome (AGBK) is characterized by cranio-facial features including macrocephaly, frontal bossing, and hypertelorism as in ACLS, and multiple epiphyseal dysplasia (MED) of upper and lower limbs which is a distinctive feature not reported in ACLS. By performing whole-exome sequencing in a family with AGBK, Ali et al. identified a homozygous missense mutation in *KIF7*, NM_198525.2:c.3179A>G p.(Asn1060Ser) [111]. Chondrocyte proliferation and differentiation in the growth plate requires proper regulation of Indian hedgehog (IHH) signaling, which is the major hedgehog ligand in chondrocytes [112]. It has been suggested that this missense mutation in KIF7 may have an abnormal function in the IHH pathway, and genetic analysis in other AGBK families is awaited.

#### 4.7.3. Hydrolethalus Syndrome 2; HLS2 #614120

Hydrolethalus syndrome is an autosomal recessive embryonic lethal disorder characterized by hydrocephaly or anencephaly, postaxial polydactyly of the upper limbs, and pre- or postaxial polydactyly of the lower limbs. Duplication of the hallux is a common finding. Putoux et al. detected three nonsense and four frameshift *KIF7* homozygous mutations in nine families with HLS2 [113]. It is noteworthy that the same p.Ala966ProfsTer81 homozygous mutation was found in both an ACLS and a HLS2 family. Although HLS2 is considered to be a severe form of ACLS, no genotype-phenotype correlation can be elucidated.

## 5. Miscellaneous Syndromes Related to SHH Pathways

### 5.1. Other Human GLI Genes

Homozygous LOF mutations in *GLI1* cause postaxial polydactyly without any other symptoms; Polydactyly, postaxial, type A8; PAPA8 #618123 [114]. Also, heterozygous LOF mutations of *GLI1* are a common finding in isolated postaxial polydactyly A/B [115]. The absence of craniofacial and central nervous system malformations may reflect the fact that GLI1 generally does not play as important a role in the SHH pathway for morphogenesis as GLI2 and GLI3. On the other hand, there is a report that a homozygous LOF mutation in *GLI1* is associated with phenotypes overlapping those of Ellis–van Creveld syndrome—not only postaxial polydactyly but shortening of the lower extremities [116]. Moreover, there are cases in which a homozygous missense mutation in *GLI1*, p.Leu506Gln, presents with thumb polydactyly; Polydactyly, preaxial I; PPD1 #174400 [117], but the details of the functional consequences of this mutation are not well established. There are no known human phenotypes associated with *GLI4* mutations.

### 5.2. Other Human Hedgehog Genes

A heterozygous *IHH* missense mutation (p.Asp100Asn [118]) and an in-frame deletion (p.del95E [119]) were reported in Brachydactyly, type A1; BDA1 #112500. BDA1 comprises hypoplasia/aplasia of the middle phalanges with or without symphalangism of the middle and the proximal phalanges. Homozygous *IHH* missense mutations—p.Pro46Leu in one family and p.Val190Ala in another family—were reported in Acrocapitofemoral dysplasia; ACFD #607778 [120], clinically characterized by short limbed dwarfism, short hand with brachydactyly, and narrow thorax. Experiments in a mouse model showed that IHH signaling controls growth of bones by coordinating chondrocyte proliferation and differentiation [121,122]. *IHH* mutations do not cause craniofacial abnormalities or polydactyly, suggesting that its role in morphogenesis is different from that of SHH.

The function of DHH is mainly specialized in the formation of gonads. Homozygous *DHH* LOF mutations cause 46XY sex reversal 7; SRXY7 #233420 [123], and 46XY gonadal dysgenesis with minifascicular neuropathy; GDMN #607080 [124]. Peripheral neuropathy is additionally recognized in the latter is thought to be related to the finding that expression of *Dhh* is restricted to testicular Sertoli cells and peripheral nerve Schwann cells in the developing mouse [125].

### 5.3. Ellis-Van Creveld Syndrome

Homozygous LOF mutations either *EVC* or *EVC2* cause Ellis-van Creveld syndrome; EVC #225500 [126]. EVC is characterized by narrow thorax with short ribs and postaxial polydactyly; neural tube defects do not occur and craniofacial anomalies are minimal. The EVC-EVC2 complex is required for SMO to be localized to the primary cilium when activated by SHH (Figure 1c) [13,29,30]. Deficiency of the EVC-EVC2 complex is predicted to inhibit the action of SMO and lead to a decrease in GLIA. This may explain the occurrence of postaxial polydactyly. However, craniofacial malformations are minimal, and short ribs are not observed in CJS or PHS and cannot be explained by decreased GLI activity in SHH pathway. EVC is one of the syndromes showing short-rib thoracic dysplasia (OMIM phenotypic series PS208500); the causative genes of these syndromes contain many constituent molecules of the primary cilium, but they are not always directly involved in GLI activity [127]. Interestingly, PHLS (*SMO*^−/−^) is associated with short ribs, suggesting that SMO may be associated with pathways other than GLI activity in the primary cilium.

## 6. Conclusions

The phenotypes of the major human malformation syndromes belonging to the SHH pathway are understood as a result of abnormal GLIA/R balance associated with SHH concentration in developing embryo. Notable symptoms are brain size, formation of the midface, the orientation of polydactyly with respect to the limb axis, and the development of cancer. Each syndrome is composed of a combination of these symptoms, and the occurrence of these symptoms is directly linked to the effects of mutations on the causative gene in the SHH pathway. Based on our analysis, we conclude that the characteristics of each phenotype result from whether the causal mutation leads to excess or deficiency of the total output of the SHH pathway.

## Figures and Tables

**Table 1:** Clinical Synopsis in SHH Pathway syndromes.

**Gene OMIM#**	*165230		*165240			*600725	*601309		*601500			*607035		*611254	
**Gene**	*GLI2*		*GLI3*			*SHH*	*PTCH1*		*SMO*			*SUFU*		*KIF7*	
**Location**	2q14.2		7p14.1			7q36.3	9q22.32		7q32.1			10q24.32		15q26.1	
**Phenotype OMIM#**	#615849	#610829	#175700	#174200	#146510	#142945	#109400	#610828	#241800	#601707	#109400	#617757	#200990	#607131	#614120
**Disease**	CJS	HPE9	GCPS	PAPA1	PHS	HPE3	BCNS	HPE7	PHLS	CRJS	BCNS	JBTS32	ACLS	AGBK	HLS2
**Inheritance**	AD	AD	AD	AD	AD	AD	AD	AD	AR	Mos	AD	AR	AR	AR	AR
**Mutation type**	LOF	LOF	LOF ^1^	LOF	GOF	LOF ^2^	LOF	GOF	LOF	GOF	LOF ^3^	partial LOF	LOF	LOF	LOF
**Height**	Short	Short			Short				Short			Tall	Short	Normal	
**hypopituitalism**	Y	Y			Y	Y		Y							
**Head size**	Micro	Micro	Macro			Micro	Macro	Macro	Micro		Macro	Macro	Macro	Macro	
**Holoprosencephaly**	less common	variable degree			less common	variable degree		variable degree							Anencephaly
**Intellectual disability**	some patients	Y	Normal, mild (rare)			Y	less common	Y	Speech delay	mild to moderate	less common	mild	Severe	Y	
**Eyes (telorism)**	Hypo	Hypo	Hyper	Normal	Hypo	Hypo	Hyper	Hypo			Hyper	Hyper	Hyper	Hyper	
**Microphthalmia**		Y			Y	Synophthalmia (in some)				Y					
**Mid-Face**	hypo	hypo	wide			hypo	wide	hypo			wide		wide		
**Cleft lip/palate**	both	both			both	both	both (5%)	both	Cleft palate		both (5%)		both		Cleft palate
**SMMCI**		Y				Y		Y							
**Hands Polydactyly**	Post-Ax (some)	Post-Ax	Post/Pre-Ax	Post/Pre-Ax	Post-Ax				Post-Ax	Pre-Ax		Post-Ax	Post/Pre-Ax		Post-Ax
**Hand Syndactyly**			Y	Y	Y										
**Feet polydactyly**	Post-Ax (in some)	Post-Ax	Post/Pre-Ax	Post/Pre-Ax	Post-Ax				Post-Ax	Pre-Ax (in some)		Post-Ax	Post/Pre-Ax		Post/Pre-Ax
**Feet syndactyly**			Y		Y				Y						
**Tumor**					HTH		NBCC/DMB/OC		HTH	DMB	NBCC/DMB				

^1^ Polydactyly, preaxial, type IV; PPD4 (OMIM#174700) is also caused by heterozygous *GLI3* LOF mutations. ^2^ Microphthalmia, isolated, with coloboma 5; MCOPCB5 (OMIM#611638) and Single median maxillary central incisor; SMMCI (OMIM#147250) also caused by heterozygous *SHH* LOF mutations. ^3^ Medulloblastoma, desmoplastic; MDB (OMIM#155255) and Meningioma, familial, susceptibility to (OMIM#607174) are also caused by heterozygous *SUFU* LOF mutations. ACLS: acrocallosal syndrome; AD: autosomal dominant; AGBK: Al-Gazali-Bakalinova syndrome; AR: autosomal resessive; Ax: axial; BCNS: basal cell nevus syndrome; CJS: Culler-Jones syndrome; CRJS: Curry-Jones syndrome; DMB: desmoplastic medulloblastoma; GCPS: Greig cephalopolysyndactyly syndrome; GOF: gain of function; HLS2: hydrolethalus syndrome 2; HPE: holoprosencephaly; HTH: hypothalamic hamartoma; JBTS32: Joubert syndrome 32; LOF: loss of function; NBCC: nevoid basal cell carcinoma; OC: ovarian carcinoma; PAPA1: polydactyly, postaxial, types A1 and B; PHLS: Pallister-Hall-like syndrome; PHS: Pallister-Hall syndrome; Y: yes.

## Data Availability

Not applicable.
